# Pneumococcal vaccination coverage in individuals (16–59 years) with a newly diagnosed risk condition in Germany

**DOI:** 10.1186/s12879-022-07736-1

**Published:** 2022-09-28

**Authors:** Arijita Deb, Bélène Podmore, Rosemarie Barnett, Dominik Beier, Wolfgang Galetzka, Nawab Qizilbash, Dennis Haeckl, Timo Boellinger, Kelly D. Johnson, Thomas Weiss

**Affiliations:** 1 Merck & Co., Inc, Kenilworth, NJ USA; 2 OXON Epidemiology, London, UK; 3 London School of Hygiene & Tropical Medicine, London, UK; 4 University of Bath, Bath, UK; 5 InGef-Institute for Applied Health Research Berlin GmbH, Berlin, Germany; 6 WIG2 GmbH, Leipzig, Germany; 7 MSD Sharp & Dohme GmbH, Haar, Germany

**Keywords:** Pneumococcal disease, Pneumococcal vaccination, Pneumococcal conjugate vaccine, PCV13, PPSV23, Vaccine coverage rate, Claims data, Germany

## Abstract

**Background:**

Despite recommendations from the German Standing Committee on Vaccination (STIKO), pneumococcal vaccination coverage remains low in vulnerable populations. This study estimated the pneumococcal vaccination coverage rate (VCR) and timing among individuals aged 16–59 years in Germany who were recommended to receive pneumococcal vaccination, according to STIKO.

**Methods:**

A retrospective cohort analysis was conducted using the German InGef database. Individuals aged 16 to 59 years diagnosed with at least one “at-risk” (chronic disease) or “high-risk” (e.g., immunocompromising) condition considered to be at-risk of pneumococcal infection were identified at the time of first diagnosis, between January 1, 2016 and December 31, 2018, and followed up until December 31, 2019. The percentage of cumulative pneumococcal VCR with 95% confidence interval (CI) was reported for each calendar year of follow-up.

**Results:**

There were 334,292 individuals followed for a median of 2.38 (interquartile range (IQR) 1.63–3.13) person years. For individuals aged 16–59 years diagnosed with an incident risk condition in 2016, pneumococcal VCR increased from 0.44% (95% CI 0.41–0.48) in 2016 to 1.24% (95% CI 1.18–1.30) in 2019. In 2019, VCRs were higher in individuals with high-risk conditions compared with at-risk conditions (2.24% (95% CI 2.09–2.40) vs. 0.90% (95% CI 0.85–0.96)). In 2019, VCRs were higher in individuals aged 50 to 59 years compared with individuals aged 16 to 49 years (2.25% (95% CI 2.10–2.41) vs. 0.90% (95% CI 0.84–0.96)). Similar trends were observed in individuals with newly diagnosed risk conditions identified in 2017 and in 2018. Older age, influenza vaccination and increasing number of risk conditions increased the likelihood of pneumococcal vaccination. Median time to vaccination from diagnosis of the risk condition was shorter for high-risk conditions (369.5 days (IQR 155.8–702.0)) compared to at-risk conditions (435.5 days (IQR 196.3–758.8)).

**Conclusion:**

Despite recommendations from STIKO, pneumococcal vaccination coverage remains very low and with long delays in vulnerable individuals aged 16–59 in Germany. Further efforts are required to increase immunization levels and shorten time to vaccination among individuals 16–59 years of age developing conditions with higher susceptibility to pneumococcal infection.

**Supplementary Information:**

The online version contains supplementary material available at 10.1186/s12879-022-07736-1.

## Background

Pneumococcal disease (PD) represents a leading cause of morbidity and mortality worldwide [1, 2], causing high rates of hospitalization and pressure on healthcare systems [3]. PD in the adult population affects mostly adults aged over 50 and individuals with underlying chronic conditions. The presence of chronic conditions such as chronic respiratory and cardiovascular diseases, diabetes, human immunodeficiency virus (HIV) infection or chronic renal disease has been reported to increase the risk of PD four-fold [1, 2, 4]. With the increase in chronic diseases worldwide, the morbidity and mortality caused by PD is expected to rise [4, 5]. Reports show that the global rise in associated risk conditions including diabetes, chronic respiratory and cardiovascular diseases is highest in individuals aged 45–54 years [6]. This global rise along with a growing elderly population further increases the need for vaccination against PD.

The German Standing Committee on Vaccination (“Ständige Impfkommission”, STIKO) considers individuals with underlying chronic conditions such as respiratory, cardiovascular, or metabolic disease as being “at-risk” of PD. Individuals with immunocompromising conditions or anatomical/foreign-material-associated conditions with a greater risk of meningitis are identified as “high-risk” [7]. A previous study conducted in Germany using the Institute for Applied Health Research (InGef) database (previously Health Risk Institute database) found that between 2008 and 2012, the rate of all-cause pneumonia in children and adults with at-risk conditions was 1.7 to 2.5-fold higher than for healthy controls. The rate of all-cause pneumonia in adults and children with high-risk conditions was 1.8 to 4.1-fold higher compared to healthy controls [1].

Pneumococcal vaccination recommendations vary significantly between countries, regarding eligibility and type of vaccine [8]. In Germany, national immunization schedules are reviewed yearly by STIKO, but have remained unchanged since August 2016 [7, 9]. Since 1998, STIKO has recommended pneumococcal standard vaccination in adults aged 60 and over [7]. The 23-valent pneumococcal polysaccharide vaccine (PPSV23) is currently recommended for this age group. Pneumococcal vaccination is also recommended for children, adolescents and adults with underlying medical conditions at increased risk of PD. Since 2016, for “high-risk” adults with congenital or acquired immunodeficiencies or immunosuppression, sequential vaccination with PCV13 followed by PPSV23 after 6–12 months is recommended. For “at-risk” adults, PPSV23 is recommended for those aged 16 and older. Sequential vaccination with PCV13 followed by PPSV23 after 6–12 months is recommended for “at-risk”/“high-risk” children aged 2–15 years.

Earlier studies (pre-2010) in Germany have shown the pneumococcal vaccination coverage rate (VCR) to be low in individuals aged 18–59 “at-risk” or “high-risk” for PD [10]. More recent studies (study periods of 2013–2016 [11]; 2013–2019 [12]) have demonstrated that the pneumococcal VCR remains low among individuals aged 16–59 with newly diagnosed “high-risk” immunocompromising conditions. However, the pneumococcal VCR has not been estimated in the “at-risk” adult population since before 2010.

The present study therefore aimed to provide estimates of the pneumococcal VCR between 2016 and 2019, among individuals aged 16–59 in Germany with newly diagnosed at-risk or high-risk conditions, who are not eligible for standard vaccination based on age. Time to pneumococcal vaccination and factors influencing time to pneumococcal vaccination were also explored.

## Methods

### Data source

This was a retrospective cohort study of individuals in Germany aged 16–59 years using data from a large German claims database. The InGef research database comprises de-identified longitudinal claims data from more than 9 million individuals from more than 70 statutory health insurance providers (SHIs) in Germany [13]. A sample dataset of approximately 4 million individuals who are representative of the German population for age and sex was extracted from the database and used for the present study [14]. The InGef database includes demographic information (gender, age, and region of residence), diagnostic data, mortality and morbidity data, claims data for ambulatory services and procedures, hospitalizations and drug prescription and dispensing data. Use of ambulatory services is recorded in alignment with the German uniform evaluation standard (EBM, “Einheitlicher Bewertungsmaßstab”). Procedures conducted in hospital are recorded in line with the German Procedure Classification (OPS, “Operationen und Prozedurenschlüssel”)) [14]. All diagnoses are recorded using the 10th revision of the International Classification of Diseases German Modification (ICD-10-GM).

### Study population

The source population included individuals aged 16–59 years in Germany diagnosed with an incident “at-risk” or “high-risk” condition during the study inclusion period, between January 1, 2016 and December 31, 2018. To be eligible for inclusion in the study population, all individuals had to be aged between 16 and 59 years at index date (first diagnosis of an underlying risk condition during the study inclusion period) and have at least 24 months of data available prior to study entry (study “pre-period”) to assess the presence of at-risk and high-risk conditions and pneumococcal vaccination. This was to ensure diagnoses of at-risk/high-risk conditions were incident diagnoses and to exclude individuals with prior vaccination, since repetition of pneumococcal vaccination is only recommended at intervals of at least 6 years [7]. Individuals were therefore excluded if diagnosed with an at-risk/high-risk condition before study entry; with the exception of “high-risk” patients diagnosed during the study inclusion period with an “at-risk” condition before study entry.

### Risk conditions

At-risk/high-risk conditions were defined according to the 2016/17 and 2017/18 STIKO vaccination recommendations for at-risk/high-risk individuals and availability of codes in the InGef database (Table 1) [7, 9]. The underlying medical conditions were identified by ICD-10-GM codes in the outpatient and inpatient data (all diagnosis fields) as well as OPS codes, EBM procedure codes and Anatomical Therapeutic Chemical (ATC) codes for prescriptions (see Additional file 1). The index date was defined as the first documented code for an at-risk/high-risk condition during the study inclusion period. For inpatient diagnoses, the admission date of the respective hospitalization was used to define the index date. In the outpatient data, diagnoses are recorded quarterly with no exact date. Therefore, the date of the first documented OPS- or EBM-code (e.g. dialysis to indicate chronic renal failure; organ transplant), by the diagnosing physician was used to estimate the index date.

**Table 1:** Risk condition classifications

Risk classification	Risk conditions
At-risk	Diabetes mellitus, chronic lung disease (including asthma), chronic heart disease and neurological disorders
High-risk	Cancer, chronic renal disease, functional or anatomic asplenia, sickle cell disease/other hemoglobinopathy, congenital or acquired asplenia, splenic dysfunction, splenectomy, HIV infection, immuno-compromising diseases, organ transplants, chronic liver disease and autoimmune disease Cerebrospinal fluid leak, cochlear implant

### Study design

**Fig. 1:**
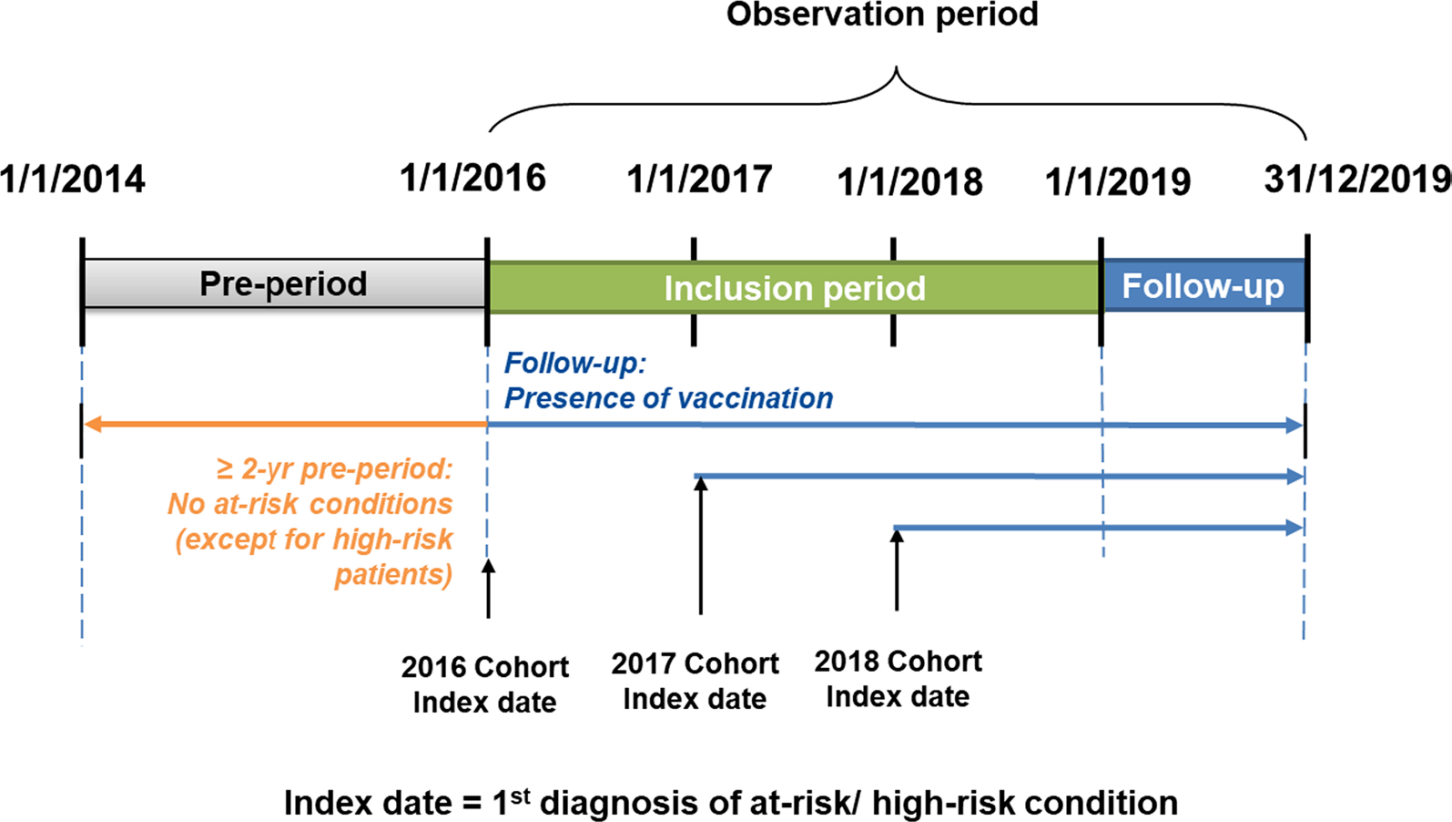
Study design

Study outcomes were assessed during the study observation period, between January 1, 2016 and December 31, 2019 (Fig. 1). A further 24 months of data (pre-period) from January 1, 2014 were assessed to ensure each adult had a minimum of 24 months preceding each individual’s study entry to identify the presence of at-risk/high-risk conditions and pneumococcal vaccination. Therefore, the first possible study entry for each adult could occur no earlier than January 1, 2016-start of the study inclusion period. The index date was defined as the date of the first diagnosis of any of the risk conditions of interest during the study inclusion period. Each individual was followed-up from the index date until the first of the following censoring criteria: end of observation in the InGef research database (based on: end of insurance with SHI contributing data to the InGef research database, end of study period (December 31, 2019), death from any cause), date of 60th birthday, or date of first pneumococcal vaccination received during the observation period.

Multiple yearly cohorts were created to assess the VCR within each calendar year of the study observation period (January 1, 2016 to December 31, 2019). The study cohorts were identified at January 1st of each calendar year from 2016 to 2018 and were followed up until December 31, 2019 (2–4 years of follow-up per cohort). For example, the 2018 cohort included all individuals newly diagnosed with an at-risk or high-risk condition during 2018; whereby 2018 comprised the first year of follow-up, 2019 the second year of follow-up. Therefore, the index date for the 2018 cohort (date of first diagnosis of an at-risk/high-risk condition within the cohort) was January 1, 2018.

### Outcomes

Pneumococcal vaccination was identified by EBM codes (89120, 89120R) in the InGef research database; capturing all reimbursed vaccinations administered in the outpatient setting by general practitioners or other specialists [11].

### Statistical methods

**Fig. 2:**
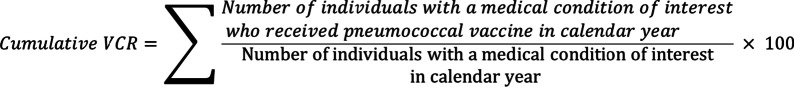
Cumulative VCR

The cumulative pneumococcal VCR (%) with 95% confidence interval (CI) was reported for each calendar year of follow-up with newly diagnosed risk conditions of interest (high-risk or at-risk) (Fig. 2). Results were presented by age-group at index date (16–49, 50–59), sex (male, female), geographic region (East, West, Berlin), first risk condition diagnosed (at-risk, high-risk), and number of risk conditions diagnosed during follow-up year. Historically, West and East Germany were classified as different federal states, with different attitudes towards vaccination; vaccination historically higher in the Eastern states [11, 15]. Hence, the decision to display regional results by East Germany, West Germany, and Berlin (East Berlin formerly part of East Germany, West Berlin formerly part of West Germany).

Time to vaccination from diagnosis was calculated with median and interquartile range (IQR). A Cox-proportional hazards model was developed to assess the factors related to time to pneumococcal vaccination: age, sex, geographic region, risk group (high-risk or at-risk), number of high-risk and at-risk conditions, month of index date (month of first diagnosis of the risk condition in the inclusion period) and influenza vaccination status. Age, number of high-risk and at-risk conditions and influenza vaccination status were time-dependent covariates in the Cox model. The factors were included in the model based on a manual backward approach; variables with evidence at the 20% level of being associated with time to pneumococcal vaccination were included in the model. The proportionality assumption was assessed with Schoenfeld residuals. Adjusted hazard ratios (HRs) and 95% Wald CIs of the final model are reported. Small numbers (< 5) were suppressed in accordance with the data source data protection policies.

## Results

**Fig. 3:**
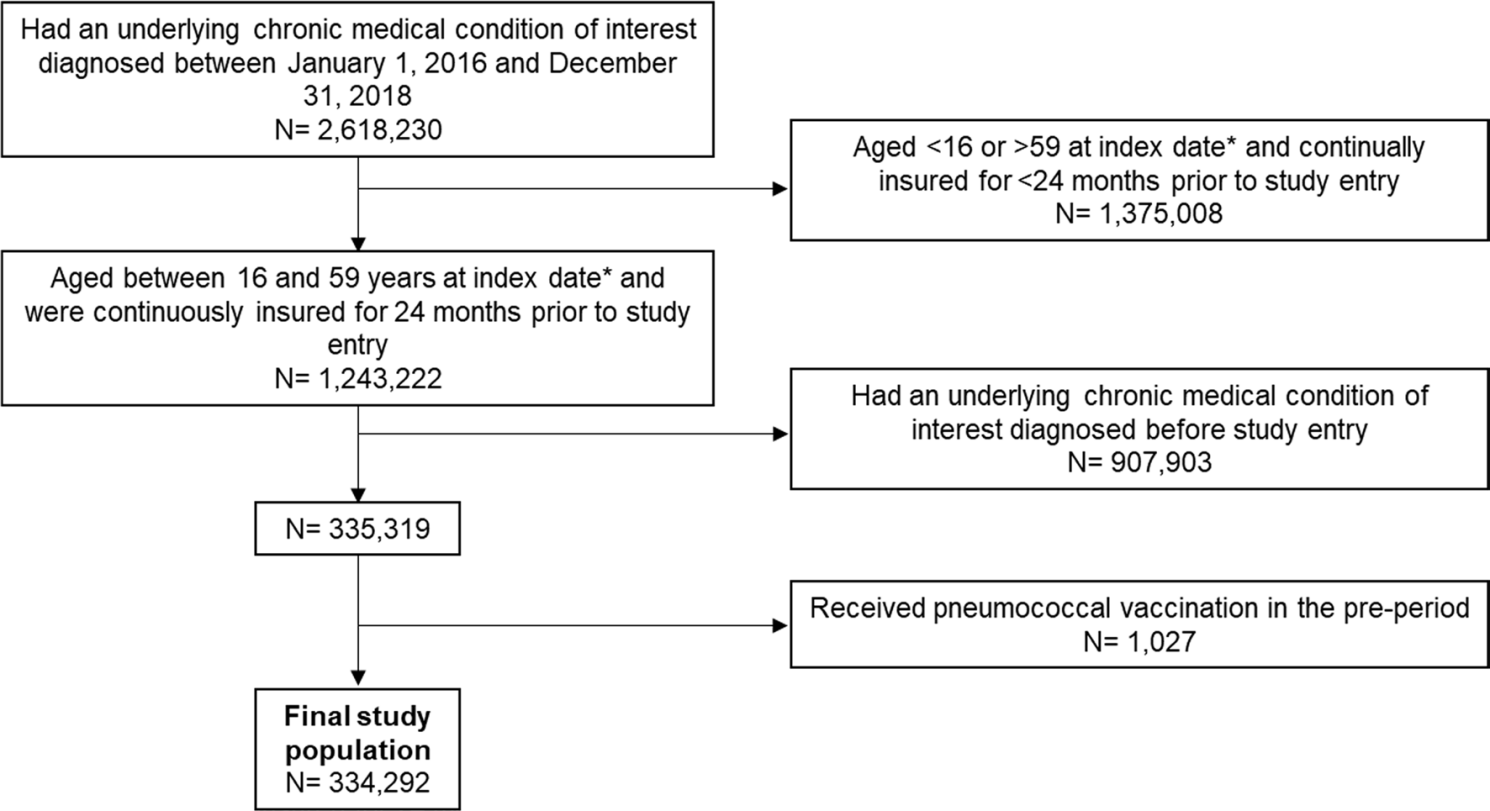
Study selection process. (Asterisk) First diagnosis of an underlying medical condition during the study inclusion period

The study population included 334,292 individuals aged 16–59 years, newly diagnosed with one or more risk condition(s) during the study inclusion period (Fig. 3; Table 2). Individuals were followed for a median of 2.38 (IQR 1.63–3.13) person years. The mean age at study entry was 39 years (standard deviation (SD) 12.36). 83.6% of the study population were newly diagnosed with an at-risk medical condition at index date. 23.5% were newly diagnosed with a high-risk medical condition at index date. The most common newly diagnosed risk conditions were the at-risk conditions chronic heart disease (38.8%) and chronic lung disease (37.3%).

**Table 2:** Baseline characteristics of the study population

Total number of individuals included in the study	334,292	
Number of individuals included in each study cohort (n, %)		
2016	138,266	41.46
2017	105,600	31.47
2018	90,426	27.07
Age (years) at study entry^a^		
Mean (SD)	39	12.36
Median (lower quartile, upper quartile)	39	29, 50
Age group (years) at study entry^a^ (n, %)		
16–49	249,575	74.66
50–59	84,717	25.34
Sex (n, %)		
Male	161,957	48.45
Female	172,335	51.55
Presence of at-risk condition at index date (n, %)		
Chronic diseases		
Diabetes mellitus	17,583	5.26
Chronic lung disease (incl. asthma)	124,870	37.35
Chronic heart disease	129,667	38.79
Neurological disorders	7313	2.19
Presence of high-risk condition at index date (n, %)		
Cancer	17,700	5.29
Cerebrospinal fluid leak	144	0.04
Chronic renal disease	6898	2.06
Cochlear implant	453	0.14
Functional or anatomic asplenia, sickle cell disease/other hemoglobinopathy, congenital or acquired asplenia, splenic dysfunction, splenectomy	1688	0.50
HIV infection	369	0.11
Immuno-compromising diseases	19,119	5.72
Organ transplant	4857	1.45
Chronic liver disease	18,851	5.64
Autoimmune disease	8494	2.54
Number of medical conditions of interest at end of follow-up^b^ in individuals with at least one at-risk medical condition		
1	261,444	78.21
2	8426	2.52
3 or more	377	0.11
Number of medical conditions of interest at end of follow-up^b^ in individuals with at least one high-risk medical condition		
1	75,804	22.68
2	1296	0.39
3 or more	58	0.02

^a^Study entry date is the latest of the following dates: January 1, 2016, the insurance start date plus 24 months, the day the individual turned 16

^b^End of follow-up = earliest of: end of observation in the InGef research database (end of insurance, end of available data and death from any cause); end of study period (December 31st, 2019); 60th birthday; first pneumococcal vaccination received during the observation period

### Vaccination coverage rates

The pneumococcal VCR among individuals aged 16–59 years who were diagnosed with an incident risk condition in 2016 (2016 cohort) increased from 0.44% (95% CI 0.41–0.48) in 2016 to 1.24% (95% CI 1.18–1.30) in 2019 (Table 3). Individuals aged 50 to 59 years were more likely to be vaccinated than those aged 16–49 years (2.25% (95% CI 2.10–2.41) vs. 0.90% (95% CI 0.84–0.96) in 2019). The VCR was highest in the Berlin region (2.04% (95% CI 1.78–2.35)) compared to both East (1.57% (95% CI 1.37–1.80)) and West (1.14% (95% CI 1.08–1.20)) Germany (2019 values presented).

Throughout the study period, the VCR was greater in individuals diagnosed with high-risk conditions than individuals diagnosed with at-risk conditions. Of the patients newly diagnosed with a risk condition in 2016, the VCR increased in both risk groups from 2016 to 2019: the group first diagnosed with an at-risk condition from 0.29% (95% CI 0.26–0.32) to 0.90% (95% CI 0.85–0.96), and group first diagnosed with a high-risk condition from 0.89% (95% CI 0.80–0.99) to 2.24% (95% CI 2.09–2.40). The VCR increased with increasing number of risk conditions diagnosed during the follow-up year. For example, in 2019, the VCR was 0.77% (95% CI 0.71–0.83) in individuals with only one at-risk condition, versus 3.76% (95% CI 3.02–4.66) in individuals with three or more at-risk conditions. For high-risk conditions, the VCR was 1.61% (95% CI 1.46–1.77) in individuals diagnosed with only one high-risk condition, versus 12.46% (95% CI 10.01–15.40) in individuals diagnosed with three or more high-risk conditions.

Similar trends were observed in the 2017 and 2018 cohorts (see Additional file 2).

**Table 3:** Pneumococcal VCR among adults with a newly diagnosed risk condition identified in 2016 (2016 cohort)

	Overall N patients with newly diagnosed risk condition in 2016	1st follow-up year 2016			2nd follow-up year 2017			3rd follow-up year 2018			4th follow-up year 2019		
		N vaccinated	VCR (%)	95% CI	N vaccinated	VCR (%)	95% CI	N vaccinated	VCR (%)	95% CI	N vaccinated	VCR (%)	95% CI
Overall	138,266	609	0.44	0.41–0.48	1037	0.75	0.71–0.80	1446	1.05	0.99–1.10	1715	1.24	1.18–1.30
Age group at index date													
16–49	103,146	310	0.30	0.27–0.34	549	0.53	0.49–0.58	770	0.75	0.70–0.80	924	0.90	0.84–0.96
50–59	35,120	299	0.85	0.76–0.95	488	1.39	1.27–1.52	676	1.92	1.79–2.07	791	2.25	2.10–2.41
Sex													
Male	66,502	302	0.45	0.41–0.51	506	0.76	0.70–0.83	692	1.04	0.97–1.12	816	1.23	1.15–1.31
Female	71,764	307	0.43	0.38–0.48	531	0.74	0.68–0.81	754	1.05	0.98–1.13	899	1.25	1.17–1.34
Geographic region													
East	12,807	65	0.51	0.40–0.65	121	0.94	0.79–1.13	170	1.33	1.14–1.54	201	1.57	1.37–1.80
West	115,921	477	0.41	0.38–0.45	800	0.69	0.64–0.74	1,112	0.96	0.90–1.02	1,321	1.14	1.08–1.20
Berlin	9438	67	0.71	0.56–0.90	116	1.23	1.03–1.47	164	1.74	1.49–2.02	193	2.04	1.78–2.35
First risk condition diagnosed at index date													
High-risk	35,102	312	0.89	0.80–0.99	503	1.43	1.31–1.56	676	1.93	1.79–2.07	785	2.24	2.09–2.40
At-risk	103,164	297	0.29	0.26–0.32	534	0.52	0.48–0.56	770	0.75	0.70–0.80	930	0.90	0.85–0.96
Number of high-risk conditions diagnosed during follow-up year^a^													
1	21,989	149	0.68	0.58–0.79	258	1.09	0.96–1.23	356	1.41	1.27–1.56	417	1.61	1.46–1.77
2	1498	44	2.94	2.20–3.92	82	3.89	3.14–4.80	129	4.68	3.95–5.53	156	5.13	4.40–5.97
3 or more	248	15	6.05	3.70–9.74	39	10.21	7.56–13.65	56	10.94	8.52–13.94	72	12.46	10.01–15.40
Number of at-risk conditions diagnosed during follow-up year^a^													
1	96,891	263	0.27	0.24–0.31	441	0.48	0.44–0.52	577	0.65	0.60–0.71	664	0.77	0.71–0.83
2	10,533	94	0.89	0.73–1.09	197	1.21	1.05–1.39	336	1.60	1.44–1.78	423	1.87	1.70–2.05
3 or more	514	7	1.36	0.66–2.78	31	2.88	2.04–4.06	61	3.40	2.65–4.34	79	3.76	3.02–4.66

N = number of patients; ^a^Denominator for VCR calculation updated for each year of follow-up, based on number of individuals diagnosed with risk conditions during each respective follow-up year

### Time to vaccination and associated factors

Of 334,292 individuals, 3,306 (0.99%) were vaccinated during the observation period (Table 4). Median time to vaccination from the first diagnosis of a high-risk condition was 369.5 (IQR 155.8–702.0) days. Median time to vaccination from the first diagnosis of an at-risk condition was 435.5 (IQR 196.3–758.8) days.

**Table 4:** Time to vaccination in individuals with one or more newly diagnosed risk condition(s) (days)

	N	N (%) vaccinated	Median	Lower quartile	Upper quartile
Any risk medical condition	334,292	3306 (0.99)	407.5	178.0	736.0
First risk condition					
High-risk	77,158	1436 (1.86)	369.5	155.8	702.0
At-risk	257,134	1870 (0.73)	435.5	196.3	758.8

Individuals aged 50–59 years were more likely to have received a pneumococcal vaccination than individuals aged 16–49 years in the study period (Table 5). Females were less likely to be vaccinated compared to males. Individuals from the Berlin region were 42% more likely to be vaccinated compared to West and East German regions. Seasonality periods were associated with vaccination, with the lowest likelihood of being vaccinated in February, May, August and November (HR 0.64 (95% CI 0.52–0.79), 0.62 (95% CI 0.50–0.78), 0.68 (95% CI 0.54–0.84) and 0.67 (95% CI 0.53–0.83), respectively compared to January). Individuals who received influenza vaccination were 4.96 (95% CI 4.59–5.36) times more likely to receive pneumococcal vaccination than those who were not vaccinated for influenza. A higher number of risk conditions also increased the likelihood of vaccination. The likelihood of vaccination was 3.82 (2.79–5.22) times higher in individuals with three or more at-risk conditions vs. one at-risk condition. The likelihood of vaccination increased further in individuals with multiple high-risk conditions; HR of 9.77 (95% CI 8.29–11.50) and 22.62 (95% 18.27–28.00) for individuals with two high-risk conditions or three or more high-risk conditions, respectively vs. one at-risk condition with no high-risk conditions.

**Table 5:** Factors associated with time to pneumococcal vaccination

	Hazard ratio	95% CI	Ρ value
Age (continuous - per year)	1.03	1.03–1.04	< 0.001
Age group at study entry			
16–49 (ref)			
50–59	1.19	1.07–1.32	< 0.001
Sex			
Male (ref)			
Female	0.91	0.85–0.98	0.009
Geographic region			
West (ref)			
East	1.05	0.95–1.17	0.345
Berlin	1.42	1.27–1.59	< 0.001
Month of vaccination			
January (ref)			
February	0.64	0.52–0.79	< 0.001
March	0.82	0.55–1.22	0.329
April	0.93	0.66–1.30	0.675
May	0.62	0.50–0.78	< 0.001
June	0.97	0.65–1.44	0.873
July	1.03	0.74–1.43	0.869
August	0.68	0.54–0.84	0.001
September	1.12	0.75–1.67	0.590
October	1.23	0.88–1.72	0.216
November	0.67	0.53–0.83	< 0.001
December	0.81	0.47–1.37	0.424
Influenza vaccination status			
Unvaccinated (ref)			
Vaccinated	4.96	4.59–5.36	< 0.001
First risk condition diagnosed			
At-risk (ref)			
High-risk	0.69	0.61–0.79	< 0.001
At-risk status			
1 at-risk condition, no high-risk condition (ref)			
2 at-risk conditions, no high-risk condition	2.40	2.14–2.69	< 0.001
3 or more at-risk conditions, no high-risk condition	3.82	2.79–5.22	< 0.001
1 high-risk condition	3.63	3.19–4.13	< 0.001
2 high-risk conditions	9.77	8.29–11.50	< 0.001
3 or more high-risk conditions	22.62	18.27–28.00	< 0.001

## Discussion

Our results indicate a very low pneumococcal VCR in individuals aged 16–59 years with a newly diagnosed risk condition in Germany. These findings are in alignment with other studies in Germany [10–12].

Theidel et al. estimated the pneumococcal VCR for the one-year period July 1, 2008 to June 30, 2009 in individuals aged 18–59 in Germany, using data from the Deutsche BKK health insurance database [10]. The VCR was estimated as 1.32% in individuals at medium risk for PD (diagnosed with a chronic cardiovascular, respiratory, metabolic, renal or neurological disorder) and 0.90% in individuals at high-risk for PD (diagnosed with an immunocompromising condition). The VCR was reported as 0.23% in individuals aged 18–59 not-at-risk of PD (i.e. not recommended for pneumococcal vaccination according to STIKO). These percentages differ from the results seen in the present study. However, the studies are difficult to compare due to differences in methodology, including the criteria for selection of the study population and risk group definitions. In contrast to the study by Theidel et al. the present study found higher VCRs in individuals with high-risk vs. at-risk conditions, as would be expected – likely explained by the different risk definitions (coding) and study inclusion criteria applied by Theidel et al., in addition to their very short study period of 1 year. Interestingly, Theidel et al. reported a higher (but still relatively low) VCR of 4.45% in adults aged 60 and over, a population who are eligible for vaccination based on older age.

Schmedt et al. investigated pneumococcal VCRs from 2014 to 2016 in adults aged 16–59 years with a newly diagnosed high-risk (immunocompromising) condition in Germany using the InGef database [11]. The authors reported a VCR of 1.6% in women, and 2.3% in men; broadly in alignment with results from the present study. Median time to vaccination was estimated at 332.5 (IQR 142–528) days (within the first two years of diagnosis) [11]—in agreement with the median time to vaccination of 369.5 (IQR 196.3–758.8) days in the present study (high-risk group). In a follow-up study to Schmedt et al., Sprenger et al. reported pneumococcal VCR within two years of a diagnosis of an incident high-risk condition, for individuals diagnosed in 2012/2013 (cohort A) versus those diagnosed in 2015–2017 (cohort B) [12]. In agreement with results reported in the present study, VCRs remained very low across both 2-year follow-up periods: estimated at 2.0% and 2.5% for cohort A and cohort B, respectively.

Increasing the VCR in individuals aged 16–59 years with newly diagnosed risk condition(s) is crucial, as there is an anticipated rise in chronic conditions diagnosed worldwide in this age group, associated with an increased risk of PD and hospitalization due to PD [5, 6, 16, 17]. A possible explanation for the low VCR and delayed time to vaccination observed in Germany could be due to the uncertainty among general practitioners and other medical professionals as to who is responsible for administering vaccinations. Schmedt et al. [11], reported that 93.2% of all vaccinations were administered by general practitioners, with similar results reported more recently by Sprenger et al. [12]. However, patients with a newly diagnosed risk condition are likely to be treated by specialists such as pneumologists, oncologists or endocrinologists and not only by general practitioners. These specialists are not all reimbursed for PD vaccinations or receive training on the STIKO vaccination guidelines and may therefore refrain from recommending vaccination. Therefore, there may be a need for raised awareness of PD-vaccination recommendations across the medical specialties who diagnose and treat patients with PD-associated risk conditions.

Furthermore, although STIKO issues general recommendations for all German federal states, there is no national immunization plan in place for pneumococcal vaccination in Germany. Some federal states even have their own vaccine committees, which can issue different recommendations to STIKO. The UK, which has a clear national immunization schedule and guidance for healthcare professionals [18] and centralized vaccine procurement, has reported much higher VCRs in at-risk populations not otherwise eligible for pneumococcal vaccination based on age. A recent UK study using data from the Clinical Practice Research Datalink reported VCRs of 13.6% in the first year of follow-up, rising to 32.0% after 4 years of follow-up between 2011 and 2015, for individuals at-risk or high-risk for PD due to underlying medical conditions [19].

Neufiend et al. recently highlighted decentralized vaccine procurement as a key contextual barrier to vaccine provision in Germany, as it makes distribution and reimbursement more challenging [20]. Interestingly, the authors reported that encouraging or incentivizing physicians to get vaccinated themselves may be an alternative avenue to improve the low adult vaccination rates in Germany. In addition to increasing institutional trust in STIKO. A survey conducted in approximately 5,000 practices in Germany has highlighted the need for automated recall/reminder-systems, implementation of routine, regular vaccination counselling and education of physician assistants to help reduce the risk of neglecting to advise patients on vaccinations [21, 20]

Our study reports a higher likelihood of pneumococcal vaccination in those who have received the influenza vaccination. Indeed, many individuals aged 16–59 for whom pneumococcal vaccination is indicated are also likely to be eligible for influenza vaccination. These two vaccines can be administered in the same session [22], and may provide a solution to increasing the pneumococcal VCR. The impact of the new coronavirus vaccination program in 2021 [23] is unknown, but may present another opportunity to increase the pneumococcal VCR.

There is currently no recommended minimum VCR for pneumococcal vaccination in Germany or internationally. Yet it is evident that there is a strong need for health policy makers to implement further clinical practice guidelines at a national level. Training of physicians and prompting at the time of diagnosis of a risk condition may also improve the VCR considerably in these vulnerable patient groups.

The main limitation of this study is the potential selection bias of the study population. The InGef database is comprised only of individuals with state health insurance and may not include all sociodemographic strata of the German population, which may have led to a biased estimation of VCR. Furthermore, due to the structure of the SHIs that provide data for the InGef research database, the proportion of insurees in the regions of East/ West Germany differs slightly from the German population; a smaller proportion of insurees residing in the eastern part of Germany for the InGef database (16.5%, versus 19.5% in the general population) [24]. Nevertheless, previous studies have demonstrated that the InGef database is representative of the German general population for age and sex, and when studying health outcomes, morbidity, and drug usage (14).

Another limitation was the inability to distinguish the different types of pneumococcal vaccines administered (e.g., PCV13 vs. PPSV23). Information on manufacturer brand is not available in the German research databases [25]. We therefore could not assess whether those individuals with an indication for sequential vaccination (based on STIKO recommendations) received PCV13 or PPSV23 or both. Further studies would benefit from using different data sources, in order to allow for the distinction between different types of vaccines (PCV13 vs. PPSV23) and to investigate delivery of sequential booster vaccinations, where indicated by STIKO. In addition, it would be useful to also assess the proportion of vaccines delivered by general practitioners versus other healthcare professionals/specialists to further understand where the pneumococcal VCR could be improved. However, Sprenger and colleagues reported that > 90% of pneumococcal vaccinations administered to immunocompromised (high-risk) patients were done so by a general practitioner, and rarely by other specialists [12].

Finally, there may be misclassification bias due to coding inaccuracies since medical conditions were identified based on administrative records. For example, individuals with history of risk conditions before the pre-period may have been classified as newly diagnosed. With only a two year look back before the index date it is possible that individuals with previous pneumococcal vaccination were misclassified as unvaccinated. The effect of this misclassification would be to underestimate pneumococcal VCRs in this population. It is possible that individuals with previously stable chronic conditions, such as asthma or diet-controlled diabetes, were misclassified as not at-risk. The effect of this misclassification would be to underestimate the eligible population for pneumococcal vaccination. However, it would be unlikely to bias the VCR in the selected population.

## Conclusion

Despite recommendations from STIKO, pneumococcal vaccination coverage remains very low and is delayed for long periods in vulnerable populations aged 16–59 years with newly diagnosed risk condition(s) in Germany. Our findings highlight that further effort is needed to improve the rate and timeliness of pneumococcal vaccination among individuals aged 16–59 years with newly diagnosed risk conditions in Germany.

## Supplementary Information

**Additional file 1: Table S1** At-risk conditions. **Table S2** High-risk conditions.

**Additional file 2**: **Table S3** Pneumococcal VCR among 16-59 year olds with a newly diagnosed risk condition identified in 2017.** Table S4** Pneumococcal VCR among 16-59 year olds with a newly diagnosed risk condition identified in 2018.

## Data Availability

The datasets generated and/or analysed during the current study are not publicly available due to the Institute for Applied Health Research Berlin GmbH (InGef, www.InGef.de) data protection policies. Access to patient-level data is not possible and all analyses must be conducted by InGef. Requests for bespoke analyses/ aggregate results should be directed to info@ingef.de, and must be reviewed and approved by InGef.

## Abbreviations

ATC: Anatomical therapeutic chemical
CI: Confidence interval
EBM: Einheitlicher Bewertungsmaßstab
HR: Hazard ratio
ICD-10-GM: 10th revision of the International Classification of Diseases German Modification
IQR: Interquartile range
N: Number of patients
OPS: Operationen und Prozedurenschlüssel
PCV13: 13-valent Pneumococcal Conjugate Vaccine
PD: Pneumococcal diseases
PPSV23: 23-valent Pneumococcal Polysaccharide Vaccine
SD: Standard deviation
SHIs: Statutory Health Insurance Providers
STIKO: The German Standing Committee on Vaccinations (“Ständige Impfkommision”)
VCR: Vaccine coverage rate

## References

[1] Pelton SI, Shea KM, Farkouh RA, Strutton DR, Braun S, Jacob C, Klok R, Gruen ES, Weycker D (2015). Rates of pneumonia among children and adults with chronic medical conditions in Germany. BMC Infect Dis.

[2] Welte T, Torres A, Nathwani D (2012). Clinical and economic burden of community-acquired pneumonia among adults in Europe. Thorac Surg Clin.

[3] Willem L, Blommaert A, Hanquet G, Thiry N, Bilcke J, Theeten H, Verhaegen J, Goossens H, Beutels P (2018). Economic evaluation of pneumococcal vaccines for adults aged over 50 years in Belgium. Hum Vacc Immunothera.

[4] Torres A, Peetermans WE, Viegi G, Blasi F (2013). Risk factors for community-acquired pneumonia in adults in Europe: a literature review. Thorac Surg Clin.

[5] World Health Organization (WHO). Global status report on noncommunicable diseases. 2020: World Health Organization; 2020.

[6] Institute for Health Metrics and Evaluation (IHME). Findings from the global burden of disease study 2017. The Lancet. 2018.

[7] German Standing Committee on Vaccination at the Robert Koch Institute. Recommendations of the Standing Committee on Vaccination (STIKO) at the Robert Koch Institute-2017/2018. Epidemiol Bull. 2017: 34.

[8] Castiglia P (2014). Recommendations for pneumococcal immunization outside routine childhood immunization programs in Western Europe. Adv Ther.

[9] German Standing Committee on Vaccination at the Robert Koch Institute. Recommendations of the Standing Committee on Vaccination (STIKO) at the Robert Koch Institute-2016/2017. Epidemiol Bull. 2016; 34.

[10] Theidel U, Kuhlmann A, Braem A (2013). Pneumococcal vaccination rates in adults in Germany: an analysis of statutory health insurance data on more than 850 000 individuals. Dtsch Arztebl Int.

[11] Schmedt N, Schiffner-Rohe J, Sprenger R, Walker J, von Eiff C, Häckl D (2019). Pneumococcal vaccination rates in immunocompromised patients—a cohort study based on claims data from more than 200,000 patients in Germany. PLoS ONE.

[12] Sprenger R, Häckl D, Kossack N, Schiffner-Rohe J, Wohlleben J, von Eiff C (2022). Pneumococcal vaccination rates in immunocompromised patients in Germany: a retrospective cohort study to assess sequential vaccination rates and changes over time. PLoS ONE.

[13] Bothe T, Walker J, Kröger C. Gender-related differences in health-care and economic costs for eating disorders: a comparative cost-development analysis for anorexia and bulimia nervosa based on anonymized claims data. Int J Eat Disord. 2021. 10.1002/eat.23610 34599621

[14] Andersohn F, Walker J (2016). Characteristics and external validity of the German Health Risk Institute (HRI) Database. Pharmacoepidemiol Drug Saf.

[15] Rehmet S, Ammon A, Pfaff G, Bocter N, Petersen LR (2002). Cross-sectional study on influenza vaccination, Germany, 1999–2000. Emerg Infect Dis.

[16] Kornum JB, Thomsen RW, Riis A, Lervang H-H, Schønheyder HC, Sørensen HT (2008). Diabetes, glycemic control, and risk of hospitalization with pneumonia: a population-based case-control study. Diabetes Care.

[17] van Hoek AJ, Andrews N, Waight PA, Stowe J, Gates P, George R, Miller E (2012). The effect of underlying clinical conditions on the risk of developing invasive pneumococcal disease in England. J Infect.

[18] Public Health England. Pneumococcal: the Green book of immunisation, Chap. 25. 2020.

[19] Matthews I, Lu X, Xia Q, Black W, Nozad B (2020). Pneumococcal vaccine coverage among individuals aged 18 to 64 years old with underlying medical conditions in the UK: a retrospective database analysis. BMC Public Health.

[20] Neufeind J, Betsch C, Habersaat KB, Eckardt M, Schmid P, Wichmann O (2020). Barriers and drivers to adult vaccination among family physicians—insights for tailoring the immunization program in Germany. Vaccine.

[21] Klett-Tammen CJ, Krause G, von Lengerke T, Castell S (2016). Advising vaccinations for the elderly: a cross-sectional survey on differences between general practitioners and physician assistants in Germany. BMC Fam Pract.

[22] Administering. Pneumococcal Vaccines. https://www.cdc.gov/vaccines/vpd/pneumo/hcp/administering-vaccine.html.

[23] COVID-19 vaccines. https://www.who.int/emergencies/diseases/novel-coronavirus-2019/covid-19-vaccines.

[24] Ludwig M, Enders D, Basedow F, Walker J, Jacob J (2022). Sampling strategy, characteristics and representativeness of the InGef research database. Public Health.

[25] Hense S, Hillebrand K, Horn J, Mikolajczyk R, Schulze-Rath R, Garbe E (2014). HPV vaccine uptake after introduction of the vaccine in Germany: an analysis of administrative data. Hum Vaccin Immunother.

