# Supplementary material for: Pneumococcal vaccination coverage in individuals (16–59 years) with a newly diagnosed risk condition in Germany
Source: BMC Infect Dis. 2022 Sep 28;22:753. doi: 10.1186/s12879-022-07736-1 (PMC9517976; doi:10.1186/s12879-022-07736-1)
Supplement: Supplementary file 1 — Additional file 1: Table S1 At-risk conditions. Table S2 High-risk conditions. [file 12879_2022_7736_MOESM1_ESM.docx]

**ADDITIONAL FILE 1**

**Code lists**

**Supplementary table 1** At-risk conditions

| **Category** | **ICD-10-GM** |
| --- | --- |
| Chronic heart disease | B26.8+I41.1, B33.2, B37.6, B58.8+I41.2, I02.0, I05, I06, I07, I08, I09, I10, I11.9, I13.0, I13.1, I13.2, I20.0, I20.1, I20.8, I20.9, I21.0, I21.1, I21.2, I21.3, I21.4, I21.9, I22, I23, I24.0, I24.1, I24.8, I24.9, I25.1, I25.2, I25.3, I25.4, I25.5, I25.6, I25.8, I25.9, I27.0, I27.1, I27.2, I27.8, I27.9, I28, I30, I31, I32, I34, I35, I36, I37, I38, I39, I40.0, I40.1, I41, I42, I43, I44, I45, I46, I47, I48, I49, I50, I51.0, I51.1, I51.2, I51.3, I51.4, I51.5, I51.7, I51.8, I51.9, I52, I97.0, I97.1, J10.82, Q20.0, Q20.1, Q20.2, Q20.3, Q20.4, Q20.5, Q20.8, Q20.9, Q21.0, Q21.1, Q21.2, Q21.3, Q21.8, Q21.9, Q22.0, Q22.1, Q22.2, Q22.3, Q22.5, Q22.9, Q23.0, Q23.1, Q23.2, Q23.3, Q23.4, Q23.8, Q24.0, Q24.1, Q24.2, Q24.3, Q24.4, Q24.5, Q24.6, Q24.8, Q24.9, Q25.1, Q25.2, R07.2, R07.3, R07.4, Z45.0, Z86.7, Z95.0, Z95.1, Z95.2, Z95.3, Z95.4, Z95.5, Z95.8 |
| Chronic lung disease | J44.0, J44.1, J44.9, J45.0, J45.1, J45.8, J45.9, E84.0, E84.11, E84.19, E84.8, E84.9, I27.2, I27.9, J40, J41, J42, J43.9, J44.8, J45.20, J45.21, J45.22, J47.1, J47.9, J60, J61, J62, J63, J64, J66, J67, J68, J69.1, J69.8, J70.1, J70.3, J70.4, J70.9, J84.01, J84.02, J84.03, J84.09, J84.1, J84.2, J84.8, J84.9, J96.10, J96.20, J98.4, P27.0, P27.1, P27.8 |
| Diabetes | E08-E13, E14, G32.8, G59.0, G63.2, K85, K86, K90.3, P70.1, R73, R81, R82.4, T38.3, Z96.4, O24.3, O24.9 |
| Neurological disorders | G04.1, G11.4, G24.0, G24.2, G40, G41, G71, G72, G80, P90, R56, G81, G82, G83.0, G83.1, G83.2, G83.3, G83.4, G83.9 |

**Supplementary table 2** High-risk conditions

| **Category** | **Code list** |
| --- | --- |
| **ICD-10-GM codes** | |
| Autoimmune diseases | M05, M06, M32, K50 |
| Cancer | C01, C02, C03, C04, C05, C06, C07, C08, C09, C10, C11, C12, C13, C14, C15, C16, C17, C18, C19, C20, C21, C22, C23, C24, C25, C26, C30, C31, C32, C33, C34, C37, C38, C39, C40, C41, C43, C45, C46, C47, C48, C49, C50, C51, C52, C53, C54, C55, C56, C57, C58, C60, C61, C62, C63, C64, C65, C66, C67, C68, C69, C70, C71, C72, C73, C74, C75, C76, C81, C82, C83, C84, C85, C86, C88, C90, C91, C92, C93, C94, C95, C96, C77, C78, C79, C80 |
| Cerebrospinal fluid leak | G96.0 |
| Chronic liver disease | B18.0, B18.1, B18.2, B19.0, B19.1, B19.2, I85.0, I85.9, K70.3, K70.4, K70.9, K71.0, K71.1, K71.3, K71.4, K71.5, K71.6, K71.7, K71.8, K71.9, K72.1, K72.9, K73.0, K73.1, K73.2, K73.8, K73.9, K74.0, K74.1, K74.2, K74.3, K74.4, K74.5, K74.6, K75.0, K75.1, K75.4, K76.1, K76.2, K76.3, K76.4, K76.5, K76.6, K76.7, K76.8, K76.9, R16.0, R16.2, Z94.4 |
| Chronic renal disease | N01, N03, N04, N05, N18, Q60, Z49, I12.0, I13.11, I13.2, N19, Z99.2 |
| Cochlear Implant | Z96.2 |
| Functional or anatomic asplenia, Sickle cell disease/other hemoglobinopathy, Congenital or acquired asplenia, Splenic dysfunction, splenectomy | D60, D61, D73.0, D73.1, D73.3, D73.4, D73.5, D73.8, Q89.01, Q89.08, Q89.3, D73.2, D73.9, D57, D56, D58, D59 |
| HIV | B20, B21, B22, B23, B24 |
| Immuno-compromising diseases | D57, D70, D71, D72, D73.0, D80, D81, D82, D83, D84, D89.1, D89.3, D89.8, D89.9, D90, M35.9, Q89.0, Z21, G97.80, Z96.2, Z94.80, Z94.81, Z94.0, Z94.1, Z94.2, Z94.3, Z94.4 |
| Organ transplant | B95.0, B95.1, B95.4, B95.5, Z94.0, Z94.1, Z94.2, Z94.3, Z44, Z94.80, Z94.81, Z94.9, Z94.88 |
| **EBM codes** | |
| Chronic renal disease | 13602, 13610, 13611, 40823-40828 |
| **OPS** | |
| Organ transplant | 5411, 8805, 5335, 5375, 5504, 5555 |
| Cochlear Implant | 52092, 52097, 52098 |
