# Supplementary material for: Pneumococcal vaccination coverage in individuals (16–59 years) with a newly diagnosed risk condition in Germany
Source: BMC Infect Dis. 2022 Sep 28;22:753. doi: 10.1186/s12879-022-07736-1 (PMC9517976; doi:10.1186/s12879-022-07736-1)
Supplement: Supplementary file 2 — Additional file 2: Table S3 Pneumococcal VCR among 16-59 year olds with a newly diagnosed risk condition identified in 2017. Table S4 Pneumococcal VCR among 16-59 year olds with a newly diagnosed risk condition identified in 2018. [file 12879_2022_7736_MOESM2_ESM.docx]

**ADDITIONAL FILE 2**

**Supplementary table 3** Pneumococcal VCR among 16-59 year olds with a newly diagnosed risk condition identified in 2017

|  | **Overall N patient with newly diagnosed risk condition in 2017** | **1^st^ follow-up year**  **2017** | | | **2^nd^ follow-up year**  **2018** | | | **3^rd^ follow-up year**  **2019** | | | |
| --- | --- | --- | --- | --- | --- | --- | --- | --- | --- | --- | --- |
|  |  | **N vaccinated** | **VCR (%)** | **95% CI** | **N vaccinated** | **VCR (%)** | **95% CI** | **N**  **vaccinated** | | **VCR (%)** | **95% CI** |
| **Overall** | 105600 | 480 | 0.45 | 0.42-0.50 | 820 | 0.78 | 0.73-0.83 | 986 | 0.93 | | 0.88-0.99 |
| **Age group at index date** |  |  |  |  |  |  |  |  |  | |  |
| 16-49 | 78994 | 255 | 0.32 | 0.29-0.36 | 439 | 0.56 | 0.51-0.61 | 528 | 0.67 | | 0.61-0.73 |
| 50-59 | 26606 | 225 | 0.85 | 0.74-0.96 | 381 | 1.43 | 1.30-1.58 | 458 | 1.72 | | 1.57-1.88 |
| **Sex** |  |  |  |  |  |  |  |  |  | |  |
| Male | 51378 | 251 | 0.49 | 0.43-0.55 | 403 | 0.78 | 0.71-0.86 | 487 | 0.95 | | 0.87-1.04 |
| Female | 54222 | 229 | 0.42 | 0.37-0.48 | 417 | 0.77 | 0.70-0.85 | 499 | 0.92 | | 0.84-1.00 |
| **Geographic region** |  |  |  |  |  |  |  |  |  | |  |
| East | 10003 | 58 | 0.58 | 0.45-0.75 | 103 | 1.03 | 0.85-1.25 | 128 | 1.28 | | 1.08-1.52 |
| West | 88038 | 373 | 0.42 | 0.38-0.47 | 628 | 0.71 | 0.66-0.77 | 744 | 0.85 | | 0.79-0.91 |
| Berlin | 7527 | 49 | 0.65 | 0.49-0.86 | 89 | 1.18 | 0.96-1.45 | 114 | 1.51 | | 1.26-1.82 |
| **First risk condition diagnosed** |  |  |  |  |  |  |  |  |  | |  |
| High-risk | 23035 | 220 | 0.96 | 0.84-1.09 | 345 | 1.50 | 1.35-1.66 | 398 | 1.73 | | 1.57-1.90 |
| At-risk | 82565 | 260 | 0.31 | 0.28-0.36 | 475 | 0.58 | 0.53-0.63 | 588 | 0.71 | | 0.66-0.77 |
| **Number of high-risk conditions diagnosed during follow-up year** | | | | | | | | | | | |
| 1 | 17806 | 140 | 0.79 | 0.67-0.93 | 236 | 1.24 | 1.09-1.40 | 272 | 1.39 | | 1.23-1.56 |
| 2 | 1238 | 52 | 4.20 | 3.22-5.47 | 93 | 5.38 | 4.41-6.55 | 108 | 5.56 | | 4.63-6.67 |
| 3 or more | 220 | 13 | 5.91 | 3.49-9.85 | 42 | 12.14 | 9.11-16.00 | 54 | 13.50 | | 10.50-17.20 |
| **Number of at-risk conditions diagnosed during follow-up year** | | | | | | | | | | | |
| 1 | 77870 | 235 | 0.30 | 0.27-0.34 | 387 | 0.52 | 0.47-0.57 | 465 | 0.64 | | 0.58-0.70 |
| 2 | 8075 | 75 | 0.93 | 0.74-1.16 | 179 | 1.45 | 1.26-1.68 | 230 | 1.66 | | 1.46-1.89 |
| 3 or more | 389 | 11 | 2.83 | 1.59-4.99 | 32 | 3.83 | 2.73-5.36 | 41 | 3.90 | | 2.89-5.25 |

**Supplementary table 4** Pneumococcal VCR among 16-59 year olds with a newly diagnosed risk condition identified in 2018

|  | **Overall N patient with newly diagnosed risk condition in 2018** | **1^st^ follow-up year**  **2018** | | | **2^nd^ follow-up year**  **2019** | | |
| --- | --- | --- | --- | --- | --- | --- | --- |
|  |  | **N vaccinated** | **VCR (%)** | **95% CI** | **N vaccinated** | **VCR (%)** | **95% CI** |
| **Overall** | 90426 | 423 | 0.47 | 0.43-0.51 | 605 | 0.67 | 0.62-0.72 |
| **Age group at index date** |  |  |  |  |  |  |  |
| 16-49 | 67435 | 222 | 0.33 | 0.29-0.38 | 325 | 0.48 | 0.43-0.54 |
| 50-59 | 22991 | 201 | 0.87 | 0.76-1.00 | 280 | 1.22 | 1.08-1.37 |
| **Sex** |  |  |  |  |  |  |  |
| Male | 44077 | 211 | 0.48 | 0.42-0.55 | 291 | 0.66 | 0.59-0.74 |
| Female | 46349 | 212 | 0.46 | 0.40-0.52 | 314 | 0.68 | 0.61-0.76 |
| **Geographic region** |  |  |  |  |  |  |  |
| East | 8701 | 50 | 0.57 | 0.44-0.76 | 70 | 0.80 | 0.64-1.02 |
| West | 75252 | 332 | 0.44 | 0.40-0.49 | 474 | 0.63 | 0.58-0.69 |
| Berlin | 6446 | 41 | 0.64 | 0.47-0.86 | 61 | 0.95 | 0.74-1.21 |
| **First risk condition diagnosed** |  |  |  |  |  |  |  |
| High-risk | 19021 | 176 | 0.93 | 0.80-1.07 | 253 | 1.33 | 1.18-1.50 |
| At-risk | 71405 | 247 | 0.35 | 0.31-0.39 | 352 | 0.49 | 0.44-0.55 |
| **Number of high-risk conditions diagnosed during follow-up year** | | | | | | | |
| 1 | 15465 | 120 | 0.78 | 0.65-0.93 | 159 | 1.00 | 0.86-1.17 |
| 2 | 1079 | 34 | 3.15 | 2.26-4.37 | 56 | 4.38 | 3.39-5.64 |
| 3 or more | 179 | 11 | 6.15 | 3.47-10.67 | 23 | 10.13 | 6.85-14.74 |
| **Number of at-risk conditions diagnosed during follow-up year** | | | | | | | |
| 1 | 67695 | 215 | 0.32 | 0.28-0.36 | 309 | 0.47 | 0.42-0.52 |
| 2 | 6614 | 72 | 1.09 | 0.87-1.37 | 108 | 1.32 | 1.10-1.59 |
| 3 or more | 321 | 11 | 3.43 | 1.92-6.03 | 21 | 4.55 | 2.99-6.85 |
